# On the Utility of Evacuations and Very Low Regimen in Certain Obscure Forms of Disease; with Cases in Illustration

**Published:** 1816-01

**Authors:** 

**Affiliations:** one of the Editors


### JOURNAL AND REVIEW.

VOL. I.] JANUARY, 1816. [NO. 1.

### PART I.

### ORIGINAL COMMUNICATIONS.

*On the Utility of Evacuations and very low Regimen in certain obscure Forms of Disease; with Cases in Illustration.* By Mr. JOHNSON, one of the Editors.

"Judicium difficile." HIPPOC.

I Believe there are few *reflecting* practitioners, who are not daily embarrassed by the presentation of anomalous and contradictory symptoms, in those *nameless* shades and forms of disease connected with, or dependent on, obscure derangements of structure or function in internal organs. It is true, that the *running* practitioner, whose greatest calculation is at what hour of the day he may be able to see such and such a case, seldom finds any difficulty in the diagnosis or treatment of the most complicated maladies; nor does the dirty work of *post mortem* examination ever harrow up any unpleasant recollections or mistakes in this man's mind! It is not from such, that I expect any attention to the following observations; though I flatter myself they will not be considered uninteresting to the thinking part of the profession.

It is a pretty general rule, among all ranks of practitioners, to direct the attention to the most prominent and distressing *symptoms,* when the nature, seat, or origin of a disease, is involved in obscurity. There is something so rational and wise in this procedure, that it may appear injudicious to controvert its propriety; but I think it would not be difficult to shew, that it frequently leads to error and to danger. When the functions or structure of any important organ, as the heart, lungs, liver, or stomach, are deranged, it cannot but follow that a defective nutrition must induce a train of symptoms characterized by *debility,* while an unequal distribution of the circulation, and irregular distribution of nervous energy, must lead to a train of anomalous symptoms, uneasy sensations, and spasmodic affections, which arrest the attention of the patient, and often of the practitioner, much more than the original source of all. Now, if we direct our remedial measures towards the *debility,* the mischief will certainly be increased, because not only the bowels will be loaded with crudities, but we shall increase the quantum of blood and other fluids in circulation, where that quantum is already too great, inasmuch as it is unequally distributed. Thus it is, that we oppress those organs, whose functions or structure are already deranged, when, by a *very low regimen* in regard to eating and drinking, and the daily removal of all disordered secretions, or pent up excretions, we should more effectually remove the apparent debility, than by all the nutritious food or tonic medicines we can prescribe.

As example is more convincing than precept, I shall relate some cases in confirmation of the foregoing reflections.

Mrs. G——, aged 30, married, and had borne one child, applied to me on the 5th of July 1815, for a complaint in her throat, which she represented as producing an undescribable sensation of constriction and pain at the root of her tongue, which gave her no rest, night or day, and rendered her life miserable. On examination of the fauces internally, nothing whatever could be perceived to indicate disease. On feeling the cornua of the os hyoides externally, I was surprised to see the right carotid artery beating out as though it lay just under the skin. On placing my finger on the artery, it appeared enlarged to at least thrice its natural calibre, as it approached the angle of the jaw; and the artery seemed to lessen in size as it was traced downwards to the chest. My attention being now excited, I learned from the unhappy woman, that she had been ill for several months, and consulted several practitioners, who treated her complaint as a nervous affection, and gave her antispasmodics with nutritious diet, recommending exercise and change of scene. She complained of much *debility*, notwithstanding this generous diet. She was very *nervous*, as she called it; easily alarmed by any sudden noise, and subject to great fluttering and *palpitation of the heart*. She had a peculiar hoarseness of voice, and a bloated countenance indicating great anxiety and distress. The pulse exhibited nothing unusual, excepting that it was rather small and weak. Her digestive organs did not appear much affected, but her appetite was not very keen. Catamenia regular, but small in quantity; rested very ill at night, and frequently disturbed with frightful dreams.

Viewing this train of symptoms as the result of some organic derangement of the heart, or great vessels issuing from thence, and regarding even the affection of the throat as entirely symptomatic, sixteen ounces of blood were abstracted from the arm; when, after a slight deliquium, this constriction and pain of the throat, which had so long tormented the patient, and puzzled the practitioners, totally went off, to the utter surprize of the sufferer, who could scarcely credit her own feelings on the occasion. I cautioned her, however, against this delusive immunity from pain, and assured her that her complaint was one of a very serious tendency, which would require the most rigorous and persevering system of self-control on her part, and a judicious application of medicine on mine. To this she fully assented, from the recollection of her former misery. A total prohibition of animal food and every kind of strong drink was enjoined. The greatest tranquillity and rest of mind and body were recommended. The bowels were kept open by aloetic medicines, and pills composed of ex. hyoscyamus, powdered squill, submuriate of mercury, and digitalis, were given morning and evening. To this was added a weekly venæsection of twelve or fifteen ounces of blood, according to circumstances.

The first night after the bleeding was the only comfortable one she had passed for six months, and for five or six days she was greatly relieved. Some recurrence of the former symptoms then took place, which another venæsection again removed in a very considerable degree. Next to the relief of pain and constriction in the throat, was the striking *increase of strength*, and elevation of spirits, which the new plan of vegetable diet and evacuations produced. This seemed inexplicable to the patient, as all her friends considered the measures which I had adopted, *in her weak low condition*, to be little else than a means of hurrying her to the grave!

In a month, the carotid artery in the neck could scarcely be discerned pulsating; and on examining it more accurately its calibre did not appear above half of what it had been when I first saw her. Her gums had been slightly affected with the mercury, and the proportion of this medicine was lowered: But she now became so anxious for venæsection, that on any little exasperation of the symptoms she requested to lose blood. She even gained flesh, and her spirits were remarkably elevated.

At this time, (1st of November, 1815,) she does not require venæsection above once in three weeks. She passes her time comfortably and tranquilly, having the utmost confidence in my power of controlling her disease, whenever it arises to any height. The artery is still considerably larger than on the other side. The palpitation of her heart is sometimes considerable, especially on the approach of the menses; once or twice a week, particularly towards the evening, she feels the sense of constriction and fulness, *but no pain* in her throat. Upon the whole, she considers herself as comparatively happy; and whatever be the precise nature of the disease, or the ultimate result, I conceive the plan of treatment has been attended with very felicitous consequences.

December 1st, 1815. On the 10th of November, from some little exertion, a considerable recurrence of the guttural constriction came on, which required the evacuation of eighteen ounces of blood from the arm, after which it immediately disappeared. The right carotid, during the constriction, beat violently, and the fauces appeared redder than usual. Since that period, she has continued in a very easy state, but cannot discontinue the pills without suffering some return of her peculiar symptoms.

Case 2.—Mrs. Hyatt, aged 32, married, but has borne no children, consulted me on the 7th of September, 1815. Her *principal* complaint was a sensation as if two cords ran down, one on each side of the neck, from the top of the head into her breast, dragging her head, as she expressed it, downwards towards the chest, and occasioning the most afflicting feelings. The next more prominent symptom was a pain in the region of the heart, shooting sometimes towards the left shoulder, but more frequently across the chest, downwards towards the right hypochondrium. She had frequent and severe "nervous head-aches," as she called them, with the most distressing depression of spirits, *debility*, and agitation of body and mind on any sudden emotion or noise. Her rest is always broken with dreams, and she feels flushes of heat pervade every part of the body very frequently: this symptom is peculiarly distressing.

On inquiry, she informed me, that these complaints had come on her a considerable time ago, at Malta, where she had resided for three years past; but that they were greatly aggravated during the prevalence of the plague in that island, when she was insulated from society, and hourly alarmed by the scenes and rumours of mortality around her. She had been treated both at Malta and Gibraltar, as a weak, nervous, irritable female; and the whole list of antispasmodic, tonic, and anodyne remedies were exhausted on her, together with the best wines and most nourishing diet, without producing the smallest good effect. Sixteen ounces of blood were taken from the arm, when she fainted. On recovering, she exclaimed, that the hitherto tormenting cords in her neck were gone; and they have not since returned. Pills, composed of ex. col. comp. sub. hyd. and antim. tart. were ordered to be taken three times a week, so as to act briskly on the bowels, and the same medicines were ordered in the intervals as in the former case. Two ounces only of animal food were permitted daily, while wine, spirits, and ale were entirely proscribed. In the course of a week, she was so much stronger than she had been for years before, that she walked a distance of five miles without being much fatigued. She slept soundly; had better spirits; was relieved considerably of the head-aches, and only felt fugitive pains, at times, in the region of the heart. Occasional symptoms, however, of a return of her complaints, forced her on *venæsection*, as the never-failing remedy; but this measure has not often been necessary, and she now passes her time cheerfully and pleasantly, rarely omiting her medicines, or deviating from the strict rules I prescribed to her, because she immediately feels the bad effects of such irregularity. The degree of confidence with which patients of this description are inspired towards their medical adviser is very gratifying, and arises out of the sudden and certain benefit which they receive from unusual and apparently contradictory means.

December 1, 1815.—With one venæsection lately, and the continued use of the opening pills, she passes her time comfortably, and even appears to increase in strength and flesh.

Case 3.—This case caused me more anxiety, doubt, and difficulty, than I ever before experienced in the treatment of any single complaint; and never was persevering exertion on the part of the prescriber, or implicit compliance on the part of the patient, more agreeably repaid by a felicitous result. J. W. a young man, 20 years of age, consulted me on the 13th of February, 1815. He had been ill about nine months, and was under the care of several practitioners, his complaint progressively increasing. He was now pale and emaciated, his countenance exhibiting a peculiarly unhealthy aspect; his pulse was quick and rather small; his skin and tongue natural. When asked what was his *principal* complaint, he answered, "shortness of breath." He had no cough; or at most, a very trifling one: but he felt as if a rope were drawn round the thorax, and tightened till he panted for breath. At night, his sufferings were indescribable: he dared not venture to lie down; and when he fell asleep, bolstered up, he started in fright and agitation that prevented his again sleeping for some hours. His next worst symptom was a pain a little below where the apex of the heart pulsates against the ribs, which pain frequently seemed to extend downwards and sidewards towards the region of the liver. There was some fulness in the epigastrium, with slight *tenderness* on pressure; but no induration or pain. His stools were always black and fœtid. He felt as if a wedge or bolt was situated across the stomach, and abridging his respiration. His appetite was not bad, but he was always troubled with flatulence after eating. The heart could be felt pulsating, but in a diffused space; he was easily agitated, and felt something in his throat resembling globus hystericus, but not often. These were all the indications I could collect.

He was bled to sixteen ounces, when he fainted. The blood exhibited a very unusual appearance, resembling milk, with a thin soft coagulum at the bottom. Pills, composed of ex. col. comp. subm. hydrarg. and ant. tart. were ordered to clear the bowels, and to keep up a constant and copious alvine discharge. A large warm plaster was applied to the breast, in which was half an ounce of ant. tartar. He was put upon a low diet, and allowed nothing but water for drink. He slept something better the two or three first nights, and the fæcal discharges were so fœtid as scarcely to be borne in the house. In a week, a copious crop of pustules under the plaster forced us to remove it, and allow them to discharge. A mixture, composed of compound infusion of gentian, with some carbonate of ammonia, was ordered three times a-day; and this plan was pursued till the 7th of March, without gaining any ground that could encourage hope. The stools continued black and fœtid; the pain in the cardiac region was not relieved; and the breathing, though something freer, was not materially altered. The only advantage was, that he slept more and started less.

I now shifted my ground: I inserted a seton over the seat of pain, and I increased the proportion of submuriate in the pills, with the view of impregnating the system; but this I found a very difficult matter to effect. At length, on the 20th of March, I was sent for in great haste, as he was represented to be extremely ill. I went with some gloomy apprehensions on my mind, but was agreeably surprised to find him in a profuse salivation, his head and jaws swelled, and incapable of taking any nourishment. On inquiry, *every vestige of his different complaints was gone!* The pain and soreness of his jaws and gums were the only grievances which remained! My feelings on this occasion may be easily conceived. I thought I had touched on the right key, and that my triumph over the disorder was complete. I believe I should have had sufficient grounds for this self gratulation, had not a sinister event shortly afterwards baffled my hopes. His stools were now, for the first time in ten months, of a yellow and natural appearance, devoid of that intolerable fœtor which had hitherto characterized them; I therefore kept up the action of the mercury on the system, though in a less degree.

Early in April, my patient's wife died in child-bed of her first infant, under circumstances of peculiar distress; and from this time, a return of the cardiac pain and thoracic constriction was evident: these symptoms, however, never amounted to any distressing degree, nor prevented the recumbent posture in bed; nor did the epigastric fulness or disordered state of the bowels ever return at all. He constantly after this, kept an open state of the bowels by the cathartic extract and calomel; and by the middle of May, he was able to make a journey to Chatham. Shortly after his return, he went to duty in this dock-yard as a shipwright. About two months ago, the constriction returned in some degree, and threatened to force him once more to give up work; but a single venæsection entirely relieved him. Since that, he has been twice bled; keeps his bowels constantly open with the pills abovementioned; and at this time, 15th of November, he has no complaint. He has gained flesh and strength, during this long course of evacuations and low regimen. His spirits are remarkably free, and excepting a slight sense of constriction *occasionally*, which venæsection *invariably removes*, he has not a single ailment.

I shall offer few speculations on this case. I think it may reasonably be viewed as a complication of hepatic and cardi-pulmonic derangement. The *latter* I conceive as symptomatic of the former;—that is, that it was sympathetic or *functional* disorder of the heart and lungs, which would very soon have become structural or organic lesion. I conceive also, that the sensation of a wedge across the stomach was owing to accumulations in the colon; but all originating in hepatic derangement.

These three cases were taken, without much selection, from a number of others, exhibiting anomalous and obscure traits, but all marked by a train of symptoms denominated *nervous*, and characterised by *debility*. They were all either cured or considerably relieved by evacuations, vascular as well as intestinal, and low diet, with aqueous drink. These cases are not meant to dazzle by their brilliancy or extraordinary danger; but they are offered with the hope of drawing the attention of the profession to a class of diseases, and to a mode of treatment, the former of which is as obscure and perplexing, as the latter has been difficult and unsuccessful. This subject will be renewed in a future Number.

J. JOHNSON.

Portsea, Dec. 6, 1815.

---

